# Laparoscopic Common Bile Duct Exploration: 9 Years Experience from a Single Center

**DOI:** 10.3389/fsurg.2016.00023

**Published:** 2016-04-25

**Authors:** Bahman Darkahi, Håkan Liljeholm, Gabriel Sandblom

**Affiliations:** ^1^Department of Surgery, Enköping Hospital, Stockholm, Sweden; ^2^Department of Clinical Sciences, Intervention and Technology (CLINTEC), Center for Digestive Diseases, Karolinska Institutet, Karolinska University Hospital, Stockholm, Sweden

**Keywords:** bile duct structure, bile duct injury, bile leakages, infections, common bile duct stones, cholangiotomy, choledochostomy

## Abstract

**Introduction:**

The aim of the study was to evaluate the safety and feasibility of laparoscopic common bile duct exploration (LCBDE) through cholangiotomy with T-tube placement in one séance for common bile duct stones (CBDS).

**Methods:**

Between January 2005 and December 2010, a total of 99 patients with CBDS stones undergoing LCBDE with T-tube insertion at Enköping Hospital, Sweden, were registered prospectively. All patients were followed up by review of the patient records according to a standardized protocol.

**Results:**

No severe intraoperative complications were registered. Four procedures required conversion to open cholecystectomy due to impacted stones or technical difficulty. The mean operative time was 194 min [(SD) 57 min]. The mean postoperative hospital stay was 4.8 days, SD 2.4 days. At secondary cholangiography, 2 (2%) retained stones were found. Two (2%) patients had minor bile leakage, which resolved spontaneously. None of the patients experienced biliary peritonitis, biliary fistula, pancreatitis, or cholangitis. No death within 30 days after surgery was seen. No patient was readmitted with clinical signs of stricture.

**Conclusion:**

If performed by a surgeon familiar with the technique, LCBDE is a safe and feasible alternative for managing CBDS. The advantages are most pronounced in the case of multiple and large CBDS. The risk for retained stones and stricture is low.

## Introduction

Since the introduction of laparoscopic cholecystectomy (LC) as a routine technique two decades ago, it has almost completely replaced open cholecystectomy as the procedure of choice for treating cholecystolithiasis. The technique has gained general acceptance for treatment of symptomatic gallstone disease the world over.

In most cases, LC can be performed uneventfully. However, a factor that may complicate the procedure is the presence of common bile duct stones (CBDS). CBDS may be diagnosed prior to the procedure or encountered unexpectedly at peroperative cholangiography. The overall prevalence of CBDS in patients undergoing LC is 4–5% (1). When performing cholecystectomy, a treatment strategy for choledocholithiasis, whether known in advance or found incidentally at peroperative cholangiography, should be prepared. Although there are numerous ways of dealing with CBDS, six approaches predominate:

(1) wait and see, leaving the stones to pass spontaneously. In cases where symptoms of retained stones develop later, postoperative endoscopic sphincterotomy is performed;
(2) transcystic laparoscopic common bile duct exploration (LCBDE). This technique is considered most suitable for stones less than 10 mm and for stones distal to the cystic duct;
(3) LCBDE through choledochotomy. This technique is most suitable for large stones in patients with common bile ducts (CBDs) wider than 10 mm. It is also the preferred approach with stones proximal to the insertion of the cystic duct;
(4) intraoperative ERC with sphincterotomy and stone extraction;
(5) postoperative ERC with sphincterotomy;
(6) conversion to open surgery and common bile duct exploration (OCSE).

Whether ERC is performed per- or postoperatively, this may be facilitated by introducing a catheter *via* the cystic duct, through the CBD and into the duodenum, enabling endoscopic sphincterotomy either per- or postoperatively.

For more than 100 years, T-tubes have been left in place after choledochotomy following CBD exploration. However, bile leakage, biliary peritonitis, and long-term postoperative stricture have been reported and may be directly associated with placement or removal of the T-tube. The severity of these complications may be underestimated. Previous studies indicate that these complications may occur more frequently and have higher morbidity and mortality than other less invasive procedures (2). The use of T-tubes has therefore been questioned, even if it is difficult to determine whether the adverse events related to the T-tubes have occurred when the T-tube was used with an optimized technique.

There are few comparative studies on the safety and effectiveness of the strategies employed for managing CBDS (3, 4). As most techniques require experience, trained staff, and adequate equipment, few centers are able to practise more than one or two of these approaches. It is thus difficult to organize randomized controlled trials comparing these techniques. Studies on the safety and efficacy of the various techniques may, however, show what may be achieved under optimal circumstances.

## Materials and Methods

**Figure 1:**
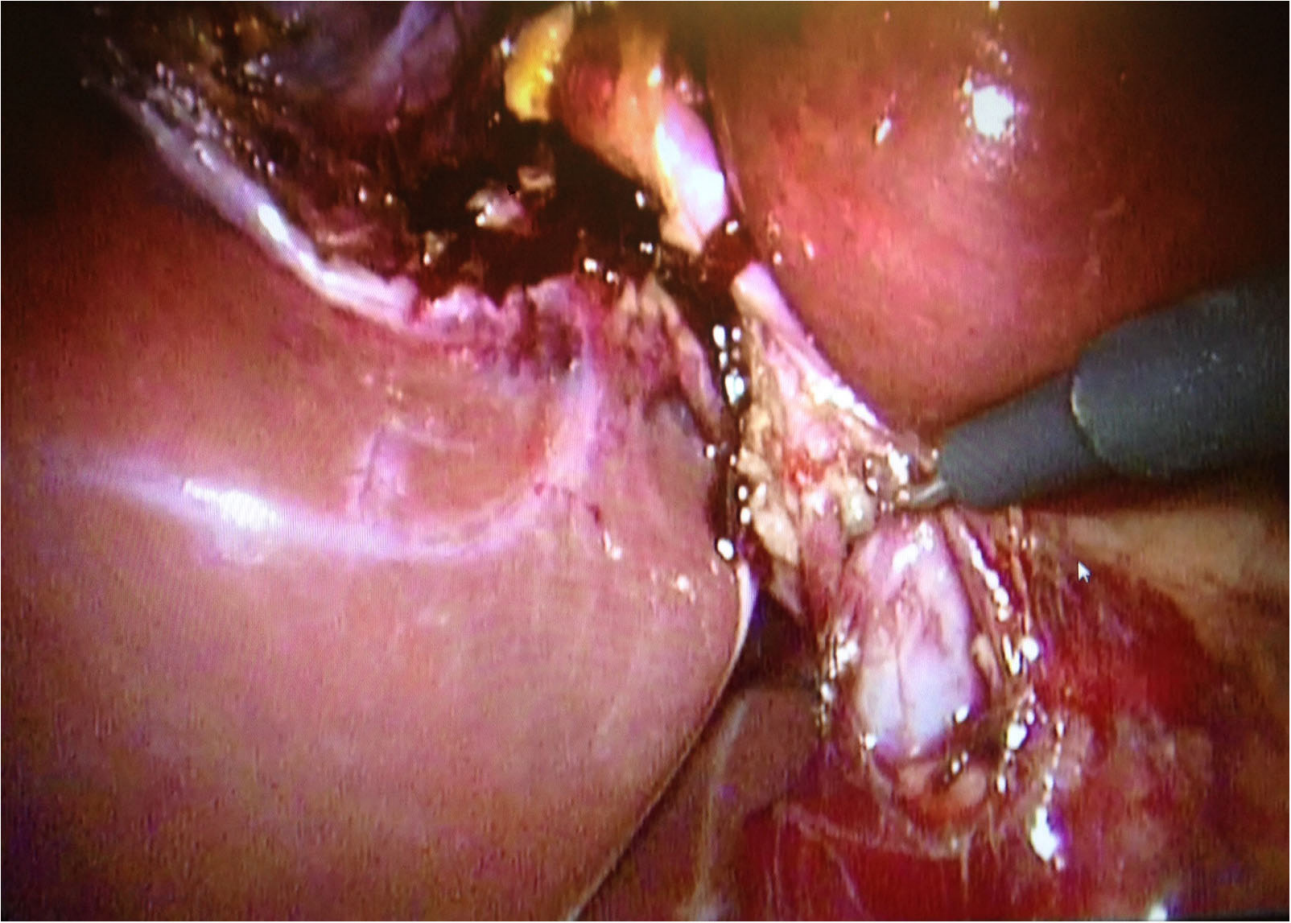
**Dissection along the common bile duct 5 mm from the junction with the cystic duct**.

**Figure 2:**
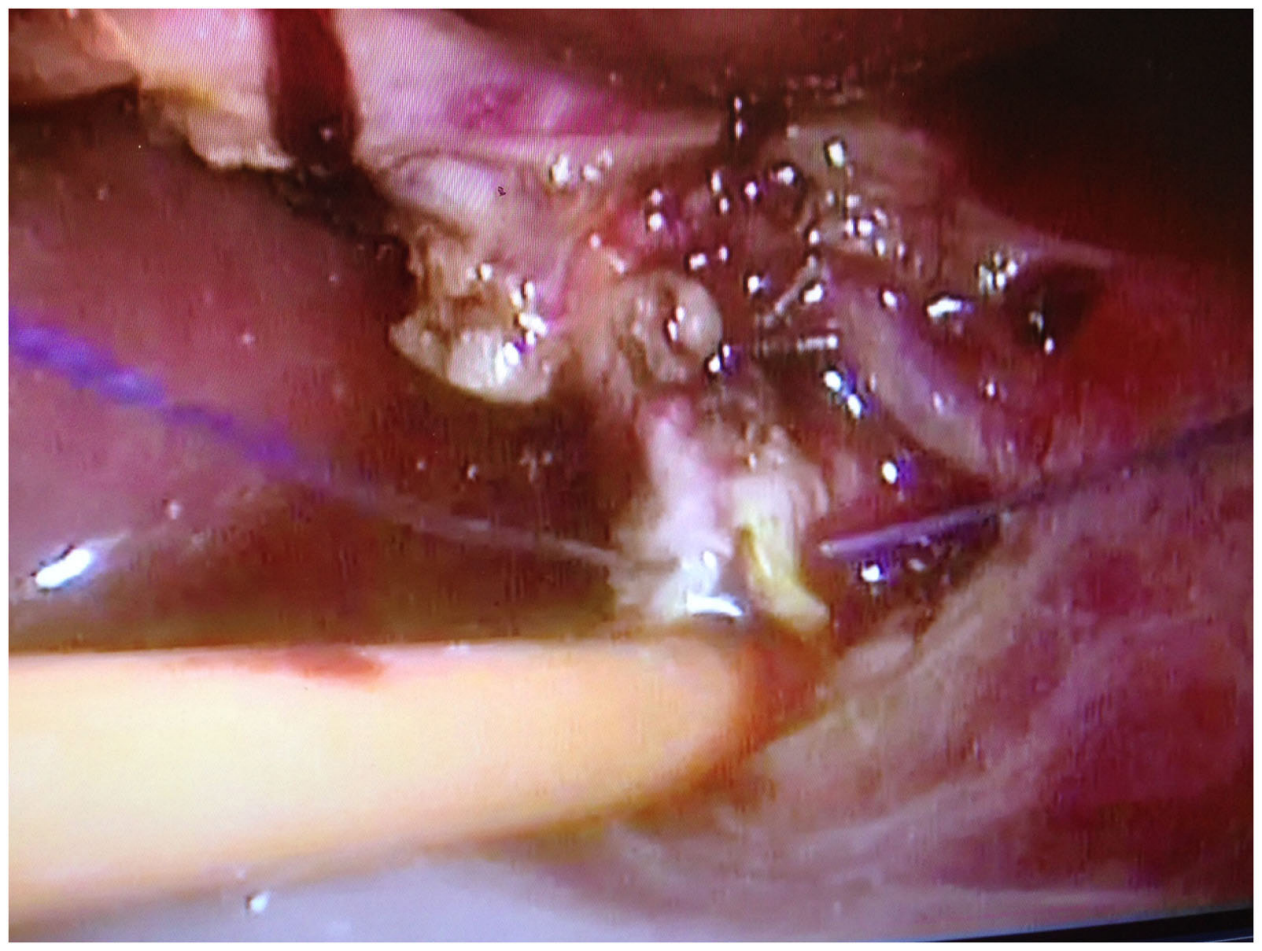
**The T-tube is inserted into the common bile duct and the incision is sutured around the T-tube**.

The present study was based on all LCBDE performed at Enköping Hospital (EH), 1995–2010. We prospectively registered all procedures with CBDS diagnosed during intraoperative cholangiography (IOC), whether diagnosed preoperatively or encountered peroperatively. The cholangiogram was assessed by a radiologist *via* direct link with the surgical theater. If calculi with a diameter of at least 5 mm were seen at peroperative cholangiography and the CDB had a minimum diameter of 6 mm, LCBDE was performed. A surgeon familiar with the technique was always present during the CBD exploration. When LBCDE was decided, 800-mg sulfamethoxazole/160-mg trimetoprim (Eusaprim^®^) was given in two doses. The LCBDE was usually performed using a four-port technique. Sometimes an extra 5 mm trocar was inserted between the subxiphoid and subcostal trocar ports. Appropriate location for the extra port was determined by inserting an 18-G needle through the abdominal wall. Choledochotomy was performed with a conventional technique, using standard laparoscopic instruments (Figure 1). The incision began 5 mm from the junction between the cystic duct and CBD. After opening the anterior peritoneal layer, peritoneum on the CBD along the free edge of the lesser omentum was divided, thereby exposing the anterior surface of the CBD. In the case of uncertainty regarding the anatomy, bile was aspirated from the CBD with a needle. The cholodocotomy incision was made vertically in the supraduodenal portion of the CBD with a retractable blade or scissors (Parrot scissors). The vessels located on either side of the CBD were avoided. The length of the incision was a maximum of 10 mm but long enough to extract all calculi. The length of the incision was documented. A 3.5 mm choledochoscope (Storz) for choledochoscopy and a dormia basket (Boston Scientific) 1.6 mm were used to extract the calculi. The number of stones extracted and the size of the largest stone were documented. During choledochoscopy, efforts were focused on achieving complete stone removal. If the surgeon did not feel entirely confident that all the stones had been removed, this was recorded in the protocol. A prefashioned T-tube, guttered along one third of its circumference lengthwise, was used, for cutting the T limb to appropriate size. The two short limbs were cut to 1 and 1.5 cm, respectively. The shorter limb was introduced toward the distal part of the CBD at a safe distance from the ampulla of Vater and the longer limb was directed upwards in order to prevent dislocation of the T-tube. The T end was introduced into the abdomen through the epigastric port. Using atraumatic graspers, the 1.5 cm arm was introduced into the CBD. The choledochotomy incision was sutured snugly around the T-tube with polyglactin 910 (Vicryl coated) 4-0 (Figure 2). After completion of the cholangiotomy, the operation was concluded by performing a cholecystectomy.

After completion of the cholecystectomy, the longer limb of the T-tube catheter was brought out through the lateral port in the abdominal wall. A passive drain (Ruche^®^, caliber 14) was applied with the tip toward the cholangiotomy. Care was taken not to dislocate the T-tube.

The T-tube was left for a period of 10 days, allowing the patient to recover. The T-tube was left open to allow bile to flow freely, thereby reducing pressure on the choledochotomy until sphincter spasm had ceased. A T-tube cholangiography was performed on the tenth postoperative day.

The T-tube was routinely clamped for 6 h on day 2 (36 h after surgery) and 24 h on day 3. During the period of clamping, the patient was monitored for pain, leakage around the tube, and fever. If none of the above features were seen, free flow of bile into the duodenum was assumed. If the T-tube cholangiography was normal, the T-tube was clamped and the passive drain removed. The T-tube was removed by gentle traction and the patient was monitored for development of abdominal signs some hours after removal. Care was taken to ensure complete removal of the horizontal limb of the T-tube, without fracturing any of the limbs. Once the tubes had been removed without complication, the patient was discharged home.

### Follow-up

The patient was invited for a clinical examination 3 months after surgery. ALT, AST, ALP, and bilirubin were controlled at follow-up. If any of these were elevated, new samples were taken 3 months later.

The study was approved by the Uppsala Ethical Review Board (Dnr 2009/239, 2009-09-30).

## Results

There were 77 women and 19 men in the study group. Data on gender were missing in three cases. Five patients could not be traced because of incorrect coding or lack of personal registration number common to the registers. Mean age was 51 years. Mean BMI was 27.1, range 17.9–40.8. A previous history of abdominal surgery was registered for 20 (19%) of the patients, but no one had undergone bariatric surgery or any other Roux-en-Y reconstruction. One patient had undergone ERCP with sphincterotomy prior to the cholecystectomy. No major peroperative complication was registered. The size of the largest stones ranged from 4 to 12 mm. Mean operative time was 194 min, in the range 75–420 min. The diameter of the CBD ranged from 8 to 20 mm. T-tubes were placed in the CBD in 37 (36%) of the procedures.

A follow-up review of the patient records was started 1 year after the last entry.

In the review of the 99 medical records, no symptoms or laboratory findings indicating bile duct complications were found. Although it cannot be excluded that some patients sought medical care outside the county of Uppsala, no symptoms or complaints that could be interpreted as late complications to the LCBDE were found in the national diagnosis register common to all hospitals in the country. No patient developed symptoms or clinical signs that could be interpreted as late stricture. The outcome of the review is presented in Table 1. Four procedures were converted to open surgery due to bleeding, severe inflammation, problems with equipment, or difficulties in introducing the T-tube. One patient underwent repeated laparoscopy after 2 days because of dislocation of the T-tube. Although bile flow was seen in the passive drain postoperatively, the amount did not exceed 100 cc/day and did not require postoperative re-operation or ERCP. At follow-up, ALT, AST, ALP, and bilirubin returned to normal in all patients. Two patients required postoperative ERCP due to retained stones seen at secondary cholangiography. Apart from that, no imaging or re-intervention was required for any of the patients.

**Table 1:** **Baseline data and outcome**.

Men	19 (19%)
Women	77 (78%)
Data missing	3 (3%)
Mean age, years (SD)	51 (18)
Previous abdominal surgery history	20 (20%)
ASA I	42 (42%)
ASA II	43 (43%)
ASA III	10 (10%)
ASA IV	1 (1%)
Number of CBDS	
1	47
2–3	16
4–7	8
>7	7
Data missing	21
Median diameter of largest stone, mm (range)	5 (4–12)
Mean operative time, min (SD)	194 (57)
Mean bleeding, ml (SD)	29 (97)
Conversion rate	4 (4%)
Mean postoperative stay, days (SD)	4.8 (2.4)
Retained stones at secondary cholangiography	2 (2%)

## Discussion

The present study shows that LCBDE can be performed safely, with no significant risk for postoperative leakage, retained stones, or bile duct stricture. This is in accordance with the findings of previous studies (5–7). The cohort presented here covers all patients undergoing cholecystectomy in EH during the study period. Now, as there are several centers performing gallstone surgery in the county of Uppsala other than the Uppsala University Hospital, the risk of selection bias is minimal. Definite conclusions regarding the technique require, however, randomized controlled trials comparing LCBDE with other approached and sufficient statistical power to detect differences in the surgical outcome.

Laparoscopic CBD exploration requires equipment and trained staff but offers advantages over many of the other methods. At present, ERCP with sphincterotomy is often considered the method of choice when managing CBDS. However, there are studies indicating that sphincterotomy may lead to late complications, including pancreatitis, bile duct stricture, and cholangitis (8). There are also studies suggesting that a sphincterotomy may predispose for pancreatic cancer and cholangiocarcinoma (9). Sphincterotomy should, thus, not be performed in patients with an expected survival long enough for late complications to develop.

Most patients prefer extraction of CBDS in one stage instead of preoperative ERC followed by LC (6). Although this may be technically more complicated, there are also other advantages to the two-stage procedure. Impacted stones may be difficult to be extracted through ERC rather than through a cholangiotomy. Furthermore, LCBDE and transcystic stone extraction can be performed in patients with a Roux-en-Y shunt (10). With the increasing numbers of patients undergoing obesity surgery, this has become an increasing challenge (11).

The mean operative time was slightly longer than 3 h, which may seem to be a relatively long time. However, most techniques for managing CBDS are time-consuming. Furthermore, the present study also included procedures performed when the technique was being introduced at the unit. As the team has gained experience, the operative time has become shorter.

In the present study, T-tubes were used in all procedures. Whether or not this is necessary has been questioned (2). In the present study, no major complication or bile leakage related to the T-tube was seen except for dislocation of the T-tube in one case. Nevertheless, the risk for problems related to the T-tube makes it necessary to carefully consider the need for the T-tube at each procedure (12). Primary closure is in many ways preferable for postoperative recovery (13, 14), but this should not be done if there is any suspicion of incomplete clearance of CBDS.

The stones extracted had, in some cases, diameters exceeding 10 mm. Stones of this size would be difficult to extract transcystically (4).

In conclusion, LCBDE is a safe procedure with little risk for stone retention. Even if it requires a trained team, experienced surgeon, and special equipment, it should be considered one of the first alternatives for managing CBDS, especially at centers with high volume. Further studies, however, are required to fully evaluate this technique. Special emphasis should be paid toward determining the risk for rare, but serious, late complications, in particular CBD stricture.

## Author Contributions

BD drafted the manuscript and assembled data. HL initiated the study, assembled data, and participated in data interpretation. GS supervised the study, performed the analyses, and helped in evaluating and editing the manuscript.

## Conflict of Interest Statement

The authors declare that the research was conducted in the absence of any commercial or financial relationships that could be construed as a potential conflict of interest.
